# Coronavirus disease 2019 (COVID-19): Multisystem review of pathophysiology

**DOI:** 10.1016/j.amsu.2021.102745

**Published:** 2021-08-23

**Authors:** Tanveer Mir, Talal Almas, Jasmeet Kaur, Mohammed Faisaluddin, David Song, Waqas Ullah, Sahil Mamtani, Hiba Rauf, Sunita Yadav, Sharaad Latchana, Nara Miriam Michaelson, Michael Connerney, Yasar Sattar

**Affiliations:** a Internal Medicine, Wayne State University, USA; b RCSI University of Medicine and Health Sciences, Dublin, Ireland; c Internal Medicine, Saint Joseph Mercy, Oakland Hospital, Pontiac, USA; d Department of Medicine, Rochester General Hospital, NY, USA; e Department of Internal Medicine, Icahn School of Medicine at Mount Sinai Elmhurst Hospital, NY, USA; f Internal Medicine, Abington Jefferson Health, PA, USA; g AtlantiCare Medical Education, 2015 Pacific Avenue, Atlantic City, NJ, USA; h Dow Medical College, Karachi, Pakistan; i NYC Health and Hospitals/Jacobi, The Bronx, NY, USA; j American University of Integrative Sciences School of Medicine, Cole Bay, Sint Maarten; k Neurology, New York Presbyterian/Weill Cornell Medical College, NY, USA; l Neurology, University of California, Los Angeles, LA, USA; m Division of Cardiology, West Virginia University, Morgantown, USA

## Abstract

Coronavirus disease-19 (COVID-19) pandemic is associated with high morbidity and mortality. COVID-19, which is caused by the Severe Acute Respiratory Syndrome Coronavirus-2 (SARS CoV-2), affects multiple organ systems through a myriad of mechanisms. Afflicted patients present with a vast constellation of symptoms, from asymptomatic disease to life-threatening complications. The most common manifestations pertain to mild pulmonary symptoms, which can progress to respiratory distress syndrome and venous thromboembolism. However, in patients with renal failure, life-threatening cardiac abnormalities can ensue. Various mechanisms such as viral entry through Angiotensin receptor (ACE) affecting multiple organs and thus releasing pro-inflammatory markers have been postulated. Nevertheless, the predictors of various presentations in the affected population remain elusive. An ameliorated understanding of the pathology and pathogenesis of the viral infection has led to the development of variable treatment options, with many more that are presently under trial. This review article discusses the pathogenesis of multiple organ involvement secondary to COVID-19 infection in infected patients.

## Highlights

• This review sheds light on the pathogenesis of multi-organ involvement secondary to COVID-19 infection.
• The predictors of various presentations in the affected population are not clear at present. A better understanding of the pathology and pathogenesis of the viral infection will be pivotal in the development of variable treatment options.
• Various proposed mechanisms such as viral entry through Angiotensin receptor (ACE) affecting multiple organs, releasing pro-inflammatory markers and cytokines inducing hypercoagulable state and cytokine storm syndrome have been postulated.

## 1 Introduction

The Coronavirus disease pandemic (COVID-19) caused by Severe Acute Respiratory Syndrome Coronavirus-2 (SARS CoV-2) has affected over 185 countries with a reported total number of 2,790,986 cases and 195,920 deaths [1]. The United States is one among the countries leading in COVID-19 associated infections and death rates [2]. The symptomatology associated with COVID-19 is variable from asymptomatic cases to multiorgan dysfunction and death [3]. Due to severe systemic inflammatory response from cytokine release, COVID-19 leads to end-organ damage and multiorgan failure (MOF) [3,4]. Understanding the underlying pathophysiology of COVID-19 for each organ system is critical for developing new therapies and improving management. We aim to do a systematic review of the literature to understand the pathophysiology of multiorgan involvement.

### 1.1 Multiorgan failure secondary to COVID-19

The multiorgan failure associated with COVID-19 is secondary to severe systemic inflammatory syndrome [3,4]. Studies have shown a strong association between ACE receptor and COVID-19 induced severe inflammatory response [5]. TMPRSS-2 is a serine protease involved in receptor-mediated endocytosis of SARS-CoV-2 [[6], [7], [8]]. The binding of SARS-CoV-2 virus results in the downregulation of ACE2 receptors, which means that angiotensin II levels begin to elevate. This elevation leads to a plethora of downstream effects on cytokine signaling, vascular homeostasis, and the coagulation cascade [5]. Cytokine storms can manifest as lymphopenia and elevated serum markers of inflammation including D-dimer, IL-6, ferritin and C-reactive protein (CRP) that can lead to multi organ failure [9].

### 1.2 Acute respiratory distress syndrome

Angiotensin-converting enzyme 2 (ACE2) receptor facilitate SARS-CoV-2 cell entry by providing a direct binding site for the S proteins of SARS-CoV-2 and promotes cleavage of angiotensin (Ang) I to produce Ang-(1–9) [10]. ACE2 receptors are widely expressed in the human body such as in nasal mucosa, bronchus, lung, heart, esophagus, kidney, stomach, bladder, and ileum which are all potential targets for COVID19 [11]. Once the virus enters, it induces ACE2 downregulation and shedding [10]. This is the primary mode of entry for SARS-COV2 and affects different organ systems. The ACE2 receptor expressed in the human airway epithelium is converted to active soluble ACE2 (sACE2) by disintegrin and metalloprotease 17.

Downregulation of ACE2 receptors is compensated by overproduction of angiotensin II (Ang II) by ACE which stimulates angiotensin II type 1a receptor that increases lung vascular permeability leading to acute lung injury and induces acute respiratory distress syndrome (ARDS) function [8]. Lung tissue has high RAS activity, enhanced during hypoxic state, and is the leading site of Ang II synthesis. Ang II is a pulmonary vasoconstrictor, resulting in pulmonary hypertension [5], and can also promote the occurrence of pulmonary edema and impair lung function [8].

In response to viral entry, the innate and adaptive immune system response secrete cytokines and inflammatory markers (see coagulopathy section) to combat the virus, specifically, increased production of cytokines interleukin-6 (IL-6), early induction of CXCL10, interleukin-2 (IL-2) and decreased production or absence of interleukin-10 (IL-10) which in turn promotes acute lung injury [12]. The viral overload and delayed type I interferon signaling further precipitate lung injury by accumulation of monocyte/macrophages that release cytokine/chemokine in the extracellular matrix and further attract accumulation of inflammatory cells induced inflammatory response and cell injury [12]. Excessive neutrophil can induce lung injury and CD8, cytotoxic T cells, contribute to lung damage from cytokines [12].

#### 1.2.1 SARS-CoV-2 coagulopathy and thromboembolism

**Fig. 1:**
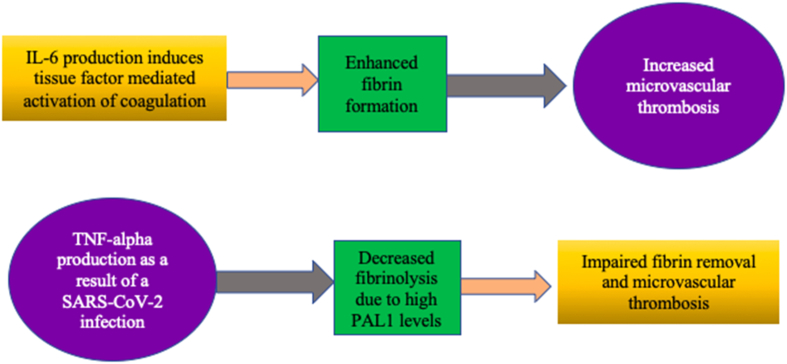
Pathogenesis of the procoagulant state secondary to COVID-19.

COVID-19 induced coagulopathy has been reported in many case reports ranging from immune thrombocytopenic purpura, deep venous thrombosis, carotid artery thrombus, pulmonary embolism, and disseminated intravascular coagulation (DIC) [10,[13], [14], [15], [16]]. Severe COVID-19 infection was associated with a more procoagulant state with higher rates of pulmonary embolism [9]. However, a meta-analysis by Mir et al. did not reveal any worsening in mortality in such critically ill patients with pulmonary embolism [17] The procoagulant state of COVID-19 infection is related with the inflammatory response of cytokines and tissue injury. Proteins expressed via SARS-CoV-2 virus likely delay the Type 1 IFN (interferon) release allowing fast viral replication, thus a dysregulated release of IFN-1 emerges [7]. As a result, proinflammatory cytokines, for example IL-6 and TNF-alpha stimulate neutrophils (PMNs) and monocytes thereby inciting a hyperinflammatory response, vascular leakage, and endothelial dysfunction [7]. Activated endothelial cells stimulate tissue factors and the extrinsic pathway whereas activated PMNs secrete neutrophil extracellular traps (NETs), which contain DAMPs that lead to activation of the intrinsic pathway via factor II. Further, combined activation of the intrinsic and extrinsic pathway coupled with reduced plasminogen activator inhibitor-1 (PAI-1) levels in ARDS resulting widespread thrombosis, a story similarly seen in the previous SARS-CoV-1 induced thrombosis [[18], [19], [20], [21]]. In addition, the upregulation of the Ang II-AT-1R axis, which promotes PAI-1 expression, the sequestration of platelets, and hypoxemia releasing a variety of hypoxia-inducible factors (HIF-1) add fuel to the impaired state [[22], [23], [24]]. High D-dimers observed in COVID-19 are representative of this dysfunctional coagulation activity requiring fibrin breakdown (Fig. 1).

#### 1.2.2 COVID and pulmonary disease

COVID-19 infection is associated with acute respiratory distress syndrome from severe inflammatory response [16]. Burst of proinflammatory cytokines initiate alveolar injury, pulmonary edema, and reduced oxygenation within pulmonary vessels. This hypoxic state leads to pulmonary vasoconstriction, increased vascular permeability with an influx of inflammatory cells within the lung parenchyma, thereby reducing surfactant levels and atelectasis. A right-to-left shunt ensues a ventilation/perfusion mismatch with an increased physiological dead space [[25], [26], [27]]. As a result of hypoxic lung injury, viral replication follows unopposed activation of Angiotensin II [28].

**Fig. 2:**
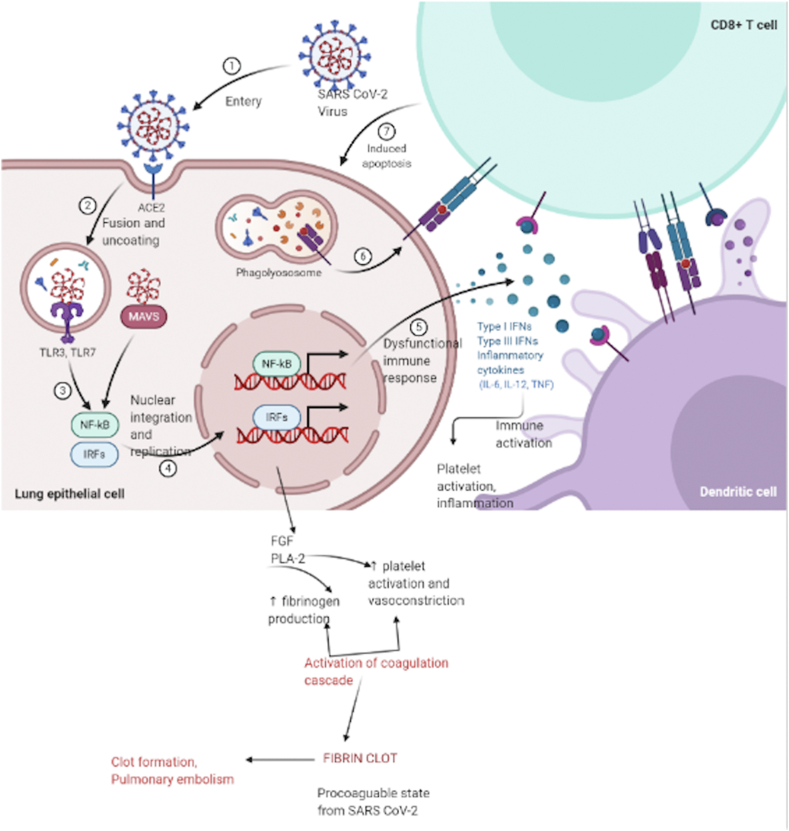
**The mechanisms underlying SARS-CoV-2 induced ARDS** [1] a hypothetical model of SARCoV-2 virus demonstrating infectious pathways to cell entery, induce inflammation, ARDS and procoagulation state. SARS CoV-2; Severe Acute Respiratory Syndrome Coronavirus-2, IL-6; Interleukin −6, TNFα; Tumor necrosis alpha, IL 1-β; Interleukin 1-beta, ACE II; Angiotensin converting enzyme II, Ang II; Angiotensin II (Created with BioRender.com).

The elevations in Ang II and decreased ACE II expression post viral entry contribute to generation of reactive oxygen species through nuclear factor kappa light chain (NFkB) activation [24] (Fig. 2). The expression of inflammatory genes further lead to generation of proinflammatory cytokines like tumor necrosis factor alpha (TNF-alpha), interleukin-1 (IL-1), and IL-6. The cytokine surge contributes to endothelial dysfunction via uncoupled nitrous oxide (eNOS), increased endothelin-1(ET-1) levels, reactive thrombocytosis, and formation of emboli which lodge into pulmonary vessels causing acute hypoxic changes [29,30]. Oxygen deficient states also cause activation of hypoxia inducible factors (HIF1-alpha, enacting angiogenesis, elevated levels of fibrinogen, and consumption of clotting factors. Further, levels of PAL-1 lead to reduced depletion of fibrin causing perfusion deficiency and pulmonary dysfunction [4,31].

### 1.3 Acute kidney injury

The mechanisms involved in heralding the onset of acute kidney injury (AKI) remain elusive, but a concoction of factors, such as hemodynamic changes, fluid overload, right heart failure, and cytokine-storm, might be at play in eliciting AKI [13]. The mechanistic features underlying AKI are therefore the subject of extensive medical research, and we hereby detail a schematic representation of the etiology underlying AKI.

**Fig. 3:**
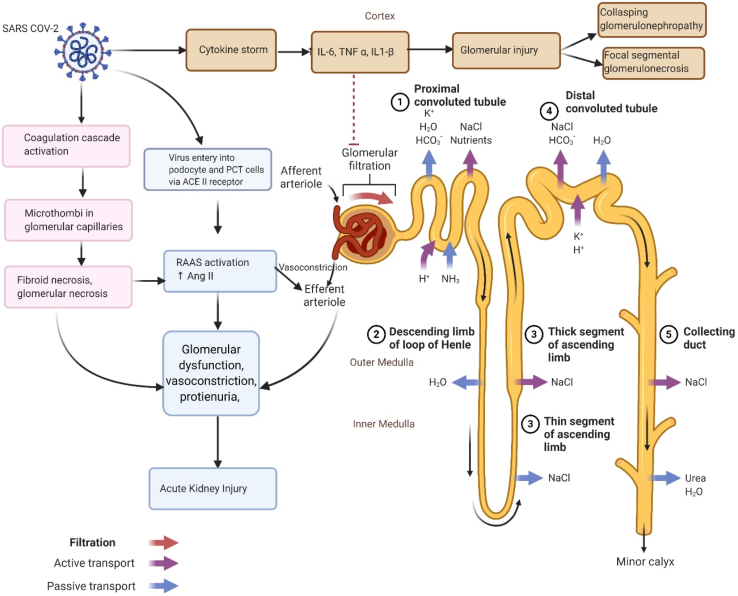
Pathogenesis underlying the acute kidney injury (AKI) seen in SARS-CoV-2. SARS CoV-2; Severe Acute Respiratory Syndrome Coronavirus-2, IL-6; Interleukin −6, TNFα; Tumor necrosis alpha, IL 1-β; Interleukin 1-beta, ACE II; Angiotensin converting enzyme II, Ang II; Angiotensin II (Created with BioRender.com).

Cytokines produced as part of a SARS-CoV-2 response, such as IL-6, upregulates the expression of sodium hydrogen exchangers. As a result, the adenosine triphosphatase (ATPase) molecules undergo hypertrophy. Complementing this tubular ATPase hypertrophy is hypoxia, which routinely results as a ramification of ARDS [25]. The hypoxia results in increased aldosterone retention, which consequently increases water retention, worsening renal failure. It is imperative to note, also, that AKI portends worse outcomes if it is superimposed on an existing chronic kidney disease (CKD) [13]. Increased acid secretion levels eventually lead to increased intraglomerular pressure, thereby damaging the filtration barrier. To compensate, tubular fibroblasts release mediators, such as NFKB and ETS-1, which culminate in tubular atrophy, inflammation, and fibrosis) [13]. The summary for mechanisms of AKI is shown in Fig. 3.

#### 1.3.1 Cardiovascular complications: heart failure, STEMI, arrhythmias, and cardiac tamponade

COVID-19 can cause cardiac complications such as myocarditis. Clinically, patients might present with chest pain, dyspnea, arrhythmias, and acute coronary syndrome (ACS) [17]. Distinguishing between ACS and myocarditis in an acute setting might be challenging. A proposed mechanism that explains myocarditis is elucidated. In addition to the mentioned cardiovascular side effects, SARS-CoV-2 also causes heart failure, pericarditis, cardiac tamponade, arrhythmias and thromboembolic events. It is presently understood that electrolyte disturbances, coupled oxidation of the Ca2+/Calmodulin-dependent protein kinase 2 is responsible for the arrhythmias seen amid a SARS-CoV-2 infection [26].

Similarly, the exorbitant increase in the production of IL-6 upregulates the expression of vascular endothelial growth factor (VEGF) resulting in increased vessel permeability and effusion that may progress to cardiac tamponade [32]. Additionally, due to deranged coagulation profiles and increased circulating levels of angiotensin II, downstream pathways involving endothelial dysfunction and oxidative stress herald the onset of thromboembolic events [3].

**Fig. 4:**
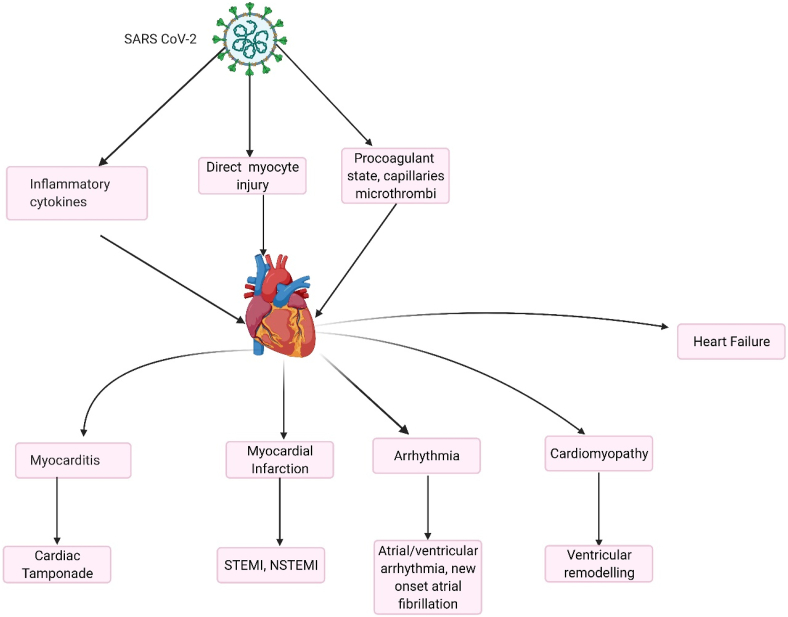
Cardiovascular pathology of COVID-19. SARS CoV-2; Severe Acute Respiratory Syndrome Coronavirus-2STEMI; ST-elevation myocardial Infarction, NSTEMI; Non-ST elevation myocardial infarction (Created with BioRender.com).

The stimulation of these pathways lead to increased production of mononuclear infiltrates, which are directed towards the myocardium, and might result in subsequent myocarditis [33]. The elevated troponin levels among patients with COVID-19 might be a result of demand ischemia rather than myocarditis itself [11]). It is therefore of clinical relevance that the troponin levels are not used exclusively, but instead in conjunction with the overall clinical picture, to yield a diagnosis of myocarditis. The graphic representation of cardiovascular pathology is in Fig. 4.

**Fig. 5:**
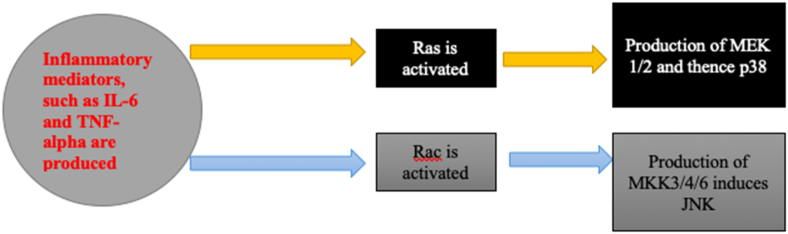
Pathogenesis of myocarditis in COVID-19 infection.

Both above detailed pathways lead to increased production of mononuclear infiltrates, which are directed towards the myocardium, leading to myocarditis [33]. Fig. 5. In study by Deng et al. troponemia in myocarditis suspect can be a demand for ischemia in the setting of COVID-19 rather than myocarditis itself [11]. It is therefore of clinical relevance that the troponin levels are not used exclusively, but instead in conjunction with the overall clinical picture, to yield a diagnosis of myocarditis.

### 1.4 Gastrointestinal

The colon and small intestine have higher expression of ACE2 receptors mainly in the endothelium and the vascular smooth muscle cells [34]. SARS-CoV-2 is associated with infiltration of plasma cells and lymphocytes in the lamina propria of stomach, duodenum and rectum that promotes interstitial edema. ACE2 receptor is also associated with neutral amino acid transporters in the gastrointestinal tract and plays an essential role. In patients with amino acid malnutrition, ACE2 promotes intestinal inflammation. ACE2 plays a significant role in amino acid homeostasis and maintaining intestinal microbiota [34]. Downregulation or alteration of ACE2 is associated with colitis, by promoting intestinal inflammation and diarrhea suggesting that SARS-Co-2 entry via ACE2 receptor may alter its activity. The proposed model is that COVID19 uses ACE2 and TMPRSS22 to enter the gut [35].

The entry of SARS-CoV-2 into the host cell begins by the binding of viral spike glycoprotein with ACE2 protein followed by processing of the spike glycoprotein by TMPRSS2 leading to membrane fusion, and recent evidence suggests an additive effect of TMPRSS4 in viral entry [35]. TMPRSS serine proteases facilitate virus infection by inducing S cleavage and exposing the fusion peptide for efficient viral entry in gastrointestinal (GI) tract [35] Inflammatory response induced by SARS-CoV-2 can also lead to direct injury of GI tract, destroying absorptive enterocytes, potentially leading to malabsorption, unbalanced intestinal secretion, and enteric nervous system [[34], [58]]. Additionally, hyperinflammation and dysregulated immune responses lead to cytokine surge which may culminate in widespread injury and serious complications like paralytic ileus and hemorrhagic colitis [22,36].

#### 1.4.1 Neurologic manifestation

Significant neurologic complications such as increased risk for ischemic and hemorrhagic stroke have become increasingly associated with COVID-19. There are many case reports and observational studies demonstrating that these patients presented with stroke often in the early stages of their illness [37,38]. Cytokine release induced hypercoagulability (as evidenced by remarkable D-Dimer elevation) contributes to significant downstream thrombus formation. Hypoxemia leading to intracellular acidosis and down-stream production of oxygen free radicals, compounded by the influx of proinflammatory cytokines, may lead to neuronal tissue ischemia. It is known that inflammation contributes significantly to atherosclerosis and increases the instability of plaques, predisposing to stroke [39].

When SARS-CoV-2 binds with ACE II receptors, patients with underlying hypertension may additionally be affected by extremes of high blood pressure, putting them at risk for intracerebral hemorrhage. Some patient cohorts have also been shown to present with thrombocytopenia, further increasing risk of intracerebral bleeding [40].

**Fig. 6:**
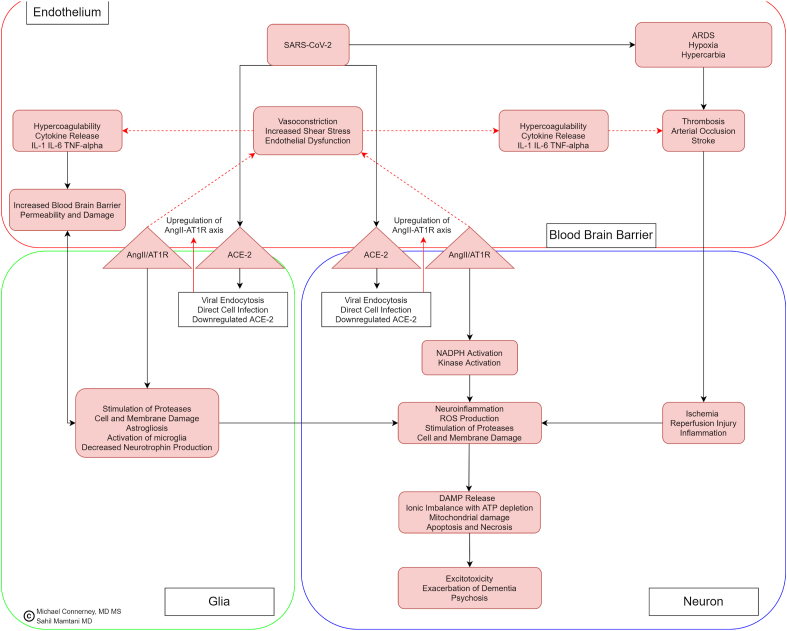
Neurologic involvement in COVID-19.

Inflammatory response and cytokine storm can affect the central nervous system by inducing toxic metabolic encephalopathy. In one confirmed case, a patient presented with fever and cough, which later progressed to acute necrotizing encephalopathy related to the cytokine storm [38]. There are also case reports of meningitis, confirmed by detection of the virus within the CSF, and hippocampal sclerosis leading to post-convulsive encephalopathy from COVID-19 [41] (Fig. 6).

SARS-CoV-2 binding to ACE-2 Receptors followed by viral endocytosis causes upregulation of Ang II/AT-1R; this results in endothelial dysfunction which includes vasoconstriction and increased shear stress. Endothelial dysfunction leads to cytokine release, including Il-1, Il-6 and TNF-alpha, culminating in increased blood brain barrier permeability. This increased inflammatory cytokine activity leads to hypercoagulability. The hypercoagulable state leading to stroke compounded by ARDS hypoxemia leads to downstream neuronal ischemia, inflammation, and necrosis secondary to reperfusion injury [[42], [43], [44], [45], [46], [47]]. Ang-II/AT1R upregulation related enzymatic activity within both glial cells and neurons, in addition to ischemia and reperfusion injury, leads to reactive oxygen species production, stimulation of proteases, with cell and membrane damage as a result [[48], [49], [50], [51], [52]]. Apoptosis of neurons occurs because of DAMP release from cell membrane damage, ATP depletion, and mitochondrial damage [[53], [54], [55], [57], [58]].

The downstream effects of this neuro-inflammatory process and excitotoxicity may manifest as altered mental status including psychosis and exacerbation of existing dementia.

### 1.5 Future directions

This review gives a potential pathophysiological mechanism behind SARS-CoV-2 infection for every system that it involves. The evidence behind these mechanisms is based on experience with similar coronaviruses and other viral infections of the same class, as well as clinical characteristics, laboratory findings, and postmortem pathological reports of COVID-19 patients around the world. The main concern is the exact contribution of risk factors to disease progression in different age, sex and race groups. Additionally, more studies are needed to understand the main drivers of COVID-19 and their molecular mechanisms of action especially in different age groups, which would help for appropriate risk stratification and therapeutic strategies. From our understanding of the published studies and evidence, immunomodulatory therapies are more likely to be equally and also very effective than just targeting virus at different stages of cycle in humans. Furthermore, treatment approaches may be further tailored to support immune response earlier during disease progression to enhance an efficient antiviral response and also to prevent progression of the disease into multi system inflammatory syndrome.

## 2 Conclusion

COVID-19 has been related as a respiratory illness, however through our extensive review of systems it can now be considered as a complex multisystem disorder. Moreover, there is a need to better understand the varied presentation of symptoms and organ involvement in different populations, nevertheless with the emerging epidemiology and basic science evidence, there has been some understanding of susceptibility of the infection and its outcomes. Even with initiation of mass vaccination drive of COVID vaccines the SARS-CoV-2 infection is expected to continue to be a burden to the healthcare sector and also to the country's economy. Hence there is need for more prospective studies to better understand this disease and varied involvements of the organs in different patients so that an effective therapy can be directed.

## Ethical approval

Research studies involving patients require ethical approval. Please state whether approval has been given, name the relevant ethics committee and the state the reference number for their judgement.

NA.

## Author contribution

TM, YS, TA: conceived the idea, designed the study, and drafted the manuscript. DS, JK, WU, SM: conducted literature search and created the illustrations. HR, SY, SL: revised the manuscript critically and refined the illustrations. TM, YS, TA, MCA: revised the final version of the manuscript critically and gave the final approval. YS conceived the idea and led this project.

## Guarantor

The Guarantor is the one or more people who accept full responsibility for the work and/or the conduct of the study, had access to the data, and controlled the decision to publish.

Talal Almas.

## Financial conflict

None.

## Declaration of competing interest

None.
